# The impact of obesity and metabolic syndrome on clinical and cognitive parameters in bipolar disorder: Results from the BIPFAT/BIPLONG study

**DOI:** 10.1192/j.eurpsy.2023.1206

**Published:** 2023-07-19

**Authors:** N. Dalkner, A. Birner, S. Bengesser, S. Guggemos, F. Fellendorf, A. Häussl, M. Lenger, A. Maget, A. Painold, M. Platzer, R. Queissner, F. Schmiedhofer, E. Schönthaler, S. Smolle, T. Stross, A. Tmava-Berisha, E. Z. Reininghaus

**Affiliations:** Psychiatry and Psychotherapeutic Medicine, Medical University Graz, Graz, Austria

## Abstract

**Introduction:**

Patients with bipolar disorder have a hig risk of becoming overweight and obese, associated with an increased risk of somatic diseases and premature mortality. The Austrian BIPFAT/BIPLONG study aims at investigating lipid metabolism, psychosocial functioning, and cognitive parameters in bipolar disorder (BD).

**Objectives:**

The aim was to investigate to what extent overweight, obesity and metabolic syndrome (MetS) are associated with clinical symptoms (e.g. suicidality, depressive symptoms) and cognitive factors (attention, memory, executive function) in BD.

**Methods:**

In addition to anamnestic interview and psychological tests, all participants were tested with a neuropsychological test battery including the Trail Making Test A/B, the Stroop Color and Word Interference Test, the d2 Test of Attention Revised, Digit Span, Digit-Symbol-Test, and the California Verbal Learning Test. Additionally, body mass index (BMI) and variables defining MetS including waist circumference, serum triglycerides, high-density lipoprotein, blood pressure, and fasting glucose levels have been collected in DSM-5 diagnosed patients with BD and healthy controls.

**Results:**

In our Austrian bipolar cohort (n=290), the median BMI was 27.9 (SD=5.9), 30.5 % of the patients were overweight (BMI = 25.5-29.9) and 24.6% of the patients were obese (BMI ≥ 30.0). In the control group (n=183), the median BMI was 24.5 (SD=4.8), 15.2% were overweight and 8.0% were obese. A sub-analysis in 215 patients showed that compared to overweight patients, normal weight patients showed more suicidal ideation in psychiatric history (χ2(2)=7.97, p=.019). In addition, there was a significant association between suicidal ideation and glucose (r=.15, p=.043) and cholesterol (r=−.17, p=.028). In another sub-analysis with 148 euthymic bipolar patients, we found a high prevalence of MetS in patients with BD (30.4% versus 15.4% in healthy controls) associated with impaired executive function compared to patients without MetS or healthy controls with and without MetS (p=.020). Clinical variables (illness duration, suicidality, number of affective episodes, medication, age of onset, and history of psychosis) did not relate to MetS in BD (p > .05). A longitudinal analysis in 52 patients (35 without MetS and 17 with MetS) did not find an association of MetS on the one-year trajectory of cognitive decline in BD. In contrast, high baseline BMI predicted a decrease in the patient’s performance in working memory in the 12-months observation period.

**Conclusions:**

The BIPFAT/BIPLONG study demonstrated a high prevalence of overweight, obesity and MetS in bipolar patients with adverse effects on cognitive function. Clinical variables such as suicidality were not related to the presence of obesity or MetS. Clinical impact and further (unpublished) results will be presented.

**Disclosure of Interest:**

None Declared

